# Incidence and long-term outcome of laser pointer maculopathy in children

**DOI:** 10.1007/s10792-023-02638-w

**Published:** 2023-01-20

**Authors:** Navid Farassat, Daniel Boehringer, Jan Luebke, Thomas Ness, Hansjuergen Agostini, Thomas Reinhard, Wolf Alexander Lagrèze, Michael Reich

**Affiliations:** Eye Center, Medical Center, Faculty of Medicine, University of Freiburg, Killianstrasse 5, 79106 Freiburg, Germany

**Keywords:** Children, Laser injury, Laser pointer, Retinal damage, Solar retinopathy

## Abstract

**Purpose:**

Single center study to evaluate the incidence and long-term outcome of laser pointer maculopathy (LPM).

**Methods:**

Medical records of 909,150 patients visiting our institution between 2007 and 2020 were screened in our electronic patient record system using the keywords "laserpointer," "laser pointer," and "solar."

**Results:**

Eight patients (6/2 male/female, 11 eyes) with a history of LPM were identified by fundoscopy and optical coherence tomography (OCT), all of whom were children (6/2 male/female). Mean age at injury was 12.1 years (range 6–16). Five children (62.5%) were injured between 2019 and 2020, three (37.5%) between 2007 and 2018. Median best-corrected visual acuity (BCVA) of affected eyes at first presentation was 20/25 (range 20/50–20/16). Follow-up examination was performed in seven children (10 eyes) with a median follow-up period of 18 months (range 0.5–96). BCVA improved in 4 children (5 eyes; BCVA at follow-up 20/22.5, range 20/40–20/16). Three of these four children were treated with oral steroids. OCT revealed acute signs such as intraretinal fluid to resolve quickly, while outer retinal disruption persisted until the last follow-up in eight of eleven eyes. These lesions resembled lesions of patients with solar retinopathy of which seven cases (11 eyes) were identified between 2007 and 2020.

**Conclusion:**

Readily available consumer laser pointers can damage the retina and the underlying retinal pigment epithelium, possibly leading to long-lasting visual impairments. The number of laser pointer injuries has increased over the last years. Therefore, access to laser pointers for children should be strictly controlled.

**Supplementary Information:**

The online version contains supplementary material available at 10.1007/s10792-023-02638-w.

## Introduction

Laser pointers play an increasingly important role as tools and toys in the modern world. However, the risks of handling laser pointers are often underestimated. In particular, vulnerable groups such as children are at risk of irreversible injury to themselves and others through irresponsible use of mostly imported and incorrectly labeled laser pointers [1, 2]. While all ocular compartments can be damaged by lasers, [3] the retina represents a predilection site due to the focusing of light onto the macula as well as the fixation behavior [4].

The risk and extent of the injury to the retina depend on the power and wavelength of the laser. Lasers are classified according to their accessible emission limits (AEL) by the IEC (International Electrotechnical Commission) 60,825 standard. Exposure to class III and IV lasers (> 1 mW) is known to have potentially detrimental effects on the eye [4, 5]. Thus, lasers are regulated in the USA by the American National Standard Institute[6] and in the European Union by the European Commission Decision of February 5, 2014, [7] allowing only the sale of up to class IIIR lasers (< 5 mW) in the USA and up to class 2 lasers (< 1 mW) in Europe [8, 9].

However, ownership and use of higher-power laser pointers are not restricted, and higher-power laser pointers are readily available on the Internet [2].

Accordingly, it is not surprising that cases of laser pointer maculopathy (LPM) have been reported with increasing frequency in recent years [10–12]. Therefore, the purpose of this study is to investigate the number of such cases in the recent past and the respective short- and long-term clinical consequences of laser pointer injuries. Thus, we aim to raise awareness for this sight-threatening condition and provide data to facilitate market regulations by national legislators.

## Methods

### Study design

In this retrospective, monocentric, observational study, we identified patients with unintended retinal laser injury, seen in the University Eye Center Freiburg, from the medical records of a total of 909,150 patients who were referred between 2007 and 2020. The keywords "laser pointer," "laserpointer" and "solar retinopathy" were used for screening in our electronic patient record system.

### Examinations

The clinical investigations performed were part of routine clinical care. Visual acuity, pupil dilation, color fundus photography (FF 450 Plus Fundus Camera, Zeiss), and spectral domain OCT (Spectralis OCT, Heidelberg Engineering) were performed in all children. Case notes including case history, fundus photography and optical coherence tomography were reviewed.

### Ethics

The study has been approved by the Central Ethics Commission in Freiburg (#21–1610) on November 4th, 2021. We comply with the Declaration of Helsinki, local laws and ICH-GCP.

### Data presentation and statistical analysis

A probability (*P*) value of < 0.05 was considered statistically significant. For the descriptive data analysis, median values and minimal and maximal values were calculated. Visual acuity is given as decimal. For comparison of BCVA improvements between children treated with oral steroids (Prednisolone 1 mg/kg body weight) and children not treated with oral steroids, we performed a two-way ANOVA test with the factors therapy (steroids or no steroids) and time of presentation (first and last presentation).

## Results

### Number of laser pointer cases from 2007 to 2020

**Fig. 1:**
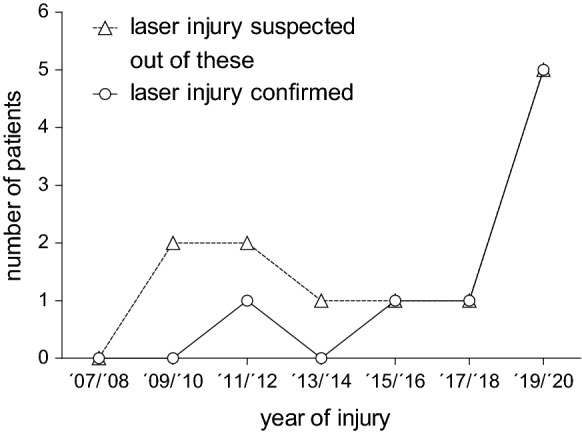
Temporal distribution of laser pointer maculopathy cases in the eye center of the university Freiburg, Germany, from 2007 to 2020. Temporal distribution of cases with suspected laser injury from laser pointers (dashed line, triangles) and cases with confirmed laser injury from laser pointers (solid line, circles)

Twelve patients with suspected laser pointer-induced retinal injuries were identified (Fig. 1, dashed line). Organic damage was confirmed in eight of these patients (11 eyes, Fig. 1, solid line). Five of these eight patients (62.5%) sustained injury in 2019/2020, while only three patients (37.5%) suffered injury between 2007 and 2018.

### Spectrum of cases with laser pointed-induced maculopathy

Detailed information of every case is presented in Table 1. Three patients were affected bilaterally, five only unilaterally. All eight patients with confirmed laser pointer-induced maculopathy (LPM) were children (6/2 male/female). The mean age at the time of injury was 12.1 years (range 6–16 years).

**Table 1:** Demographic data, laser data, symptoms, objective findings and treatment in children with laser pointer-induced retinal injury

Patient no.	Laterality of organic damage	Age at incident	Sex (m/f)	Laser color (r/g)	Laser output (mW)	Further laser information	Exposure event	Duration exposure	Symptoms	Follow-up time from initial presentation (months)	Persisting symptoms at last follow-up	BCVA at first presentation	BCVA at last presentation	Oral steroid therapy
1	OD	16	m	r	?	–	School trip	?	Central scotoma	2	Yes	20/20	20/20	Yes
2	OS	13	m	g	?	–	School trip	3–5	Blurry vision	33	Yes	20/40	20/32	Yes
3	OD OS	12	f	g	<100	Imported from bought and used in bought and used in Greece	Play with friends	5–10	Blurry vision	96	Yes	20/40 20/20	20/16 20/16	No
4	OD OS	11	m	g	<100		Play with brother	10–20	Central scotoma	18	No	20/40 20/20	20/40 20/20	No
5	OD	9	m	g	?		Play with brother	5–10	None	18	No	20/25	20/25	No
6	OD OS	14	m	?	6	–	School trip	?	Central scotoma	0.5	Yes	20/25 20/32	20/20 20/25	Yes
7	OS	6	f	r	?	Suppl. Fig 1	Play with brothers	?	Blurry vision	3	Yes	20/50	20/40	Yes
8	OD	16	m	g	<200	–	School trip	?	Blurry vision	0	NA	20/50	NA	No

In half of the cases, the retinal injuries occurred during school excursions, in the other half while playing with siblings or friends. The laser pointers emitted either red or green light. The eyes were exposed to direct laser light for 3–20 s at a distance of a few centimeters to one meter. One laser pointer could be obtained for laser power measurements (patient no. 7). This laser pointer emitted red light and had a maximum power of around 5.8 mW as measured with a laser power meter (measured with full batteries and in the immediate vicinity of the sensor; Thorlabs S130 VC), which would place the laser pointer in laser class IIIB (see Online Resource 1).

### Functional damage

Out of the eight injured children, four suffered from blurred vision and three children complained of a central scotoma in the affected eye(s). One child reported no visual deterioration. Median best-corrected visual acuity (BCVA) of affected eyes at first presentation was 20/25 (range 20/25–20/16). Follow-up examination was performed in seven children (10 injured eyes) with a median follow-up period of 18 months (range 0.5–96 months). BCVA improved in five of these eyes (median BCVA at follow-up examination 20/22.5, range 20/40–20/16). Overall, six of seven children (7 of 10 eyes) describing functional problems at initial examination described clinical residuals at follow-up that were qualitatively similar to the original symptoms but mostly milder.

### Influence of oral steroids on functional outcome

Half of the children received oral steroid therapy (Prednisolone 1 mg/kg body weight)–either for five days (three children) or tapering off over 6 weeks (one child). Visual acuity improved slightly in four of five affected eyes of children being treated with oral steroids (median BCVA at initial presentation 20/32, range 20/50–20/20; median BCVA at follow-up 20/25, range 20/40–20/20) compared with one in five affected eyes of children not treated with oral steroids (median BCVA at initial presentation 20/20, range 20/40–20/16; median BCVA at follow-up 20/20, range 20/40–20/16). BCVA improvements between first and last presentation were not significantly different between groups (two-way ANOVA: no statistically significant interaction between the effects of therapy (steroids or no steroids) and time of presentation (first and last presentation), *p* = 0.37).

### Structural damage at initial examination

**Fig. 2:**
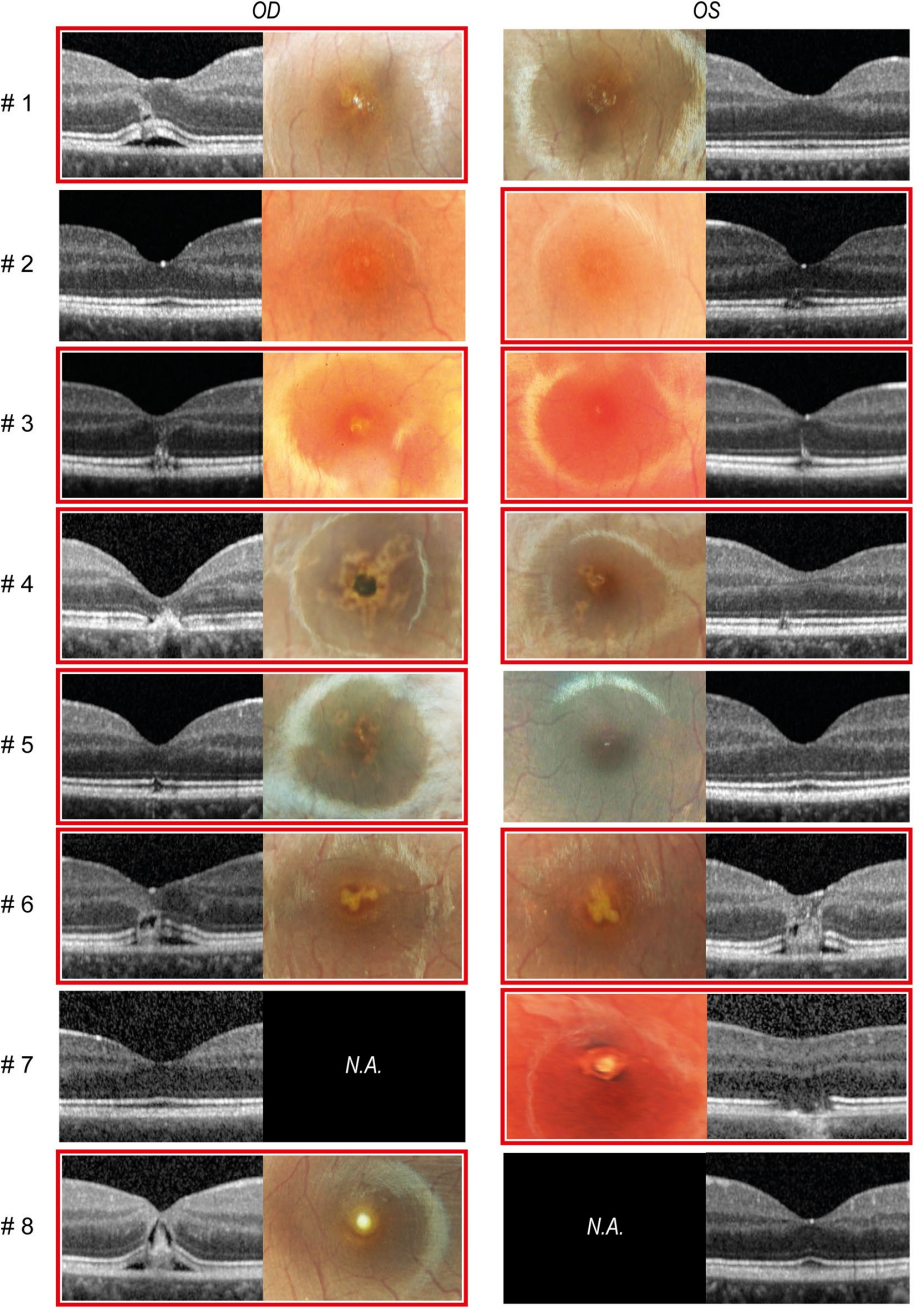
Fundoscopy and macular OCT of LPM lesions at initial presentation. Macular OCT images and fundus photography of the macula of patients with confirmed laser pointer injury. Affected eyes are marked with red borders. OD = ocular dexter, OS = ocular sinister, N.A. = not available

Slit lamp microscopy did not reveal damage to the anterior segment of the eye in any of the cases. Figure 2 shows macular OCT images and fundus photography of the macula at the initial examination of the eight patients with confirmed LPM. Fundoscopy revealed multifocal, partially confluent, yellowish-gray spots in the macula of the affected eyes. The lesions had varying phenotypes: Some were round in shape and rather small in diameter (case eye, 1 OD, 3 OD, 4 OS, 5 OD), some were likewise round but larger in diameter (7 OS, 8 OD), and others were organized as streaks (6 OD, 6 OS) or in a dendritic configuration (4 OD).

Macular OCT images of affected eyes also displayed heterogeneous lesion phenotypes ranging from hyperreflective streaks in the outer retinal layers (1 OD, 3 OD, 3 OS, 6 OD, 6 OS), disruptions of the outer retina (all affected eyes), intraretinal and subretinal fluid (1 OD, 6 OD, 6 OS,8 OD). All affected eyes showed loss of the ellipsoid zone.

### Time course of the structural damage

**Fig. 3:**
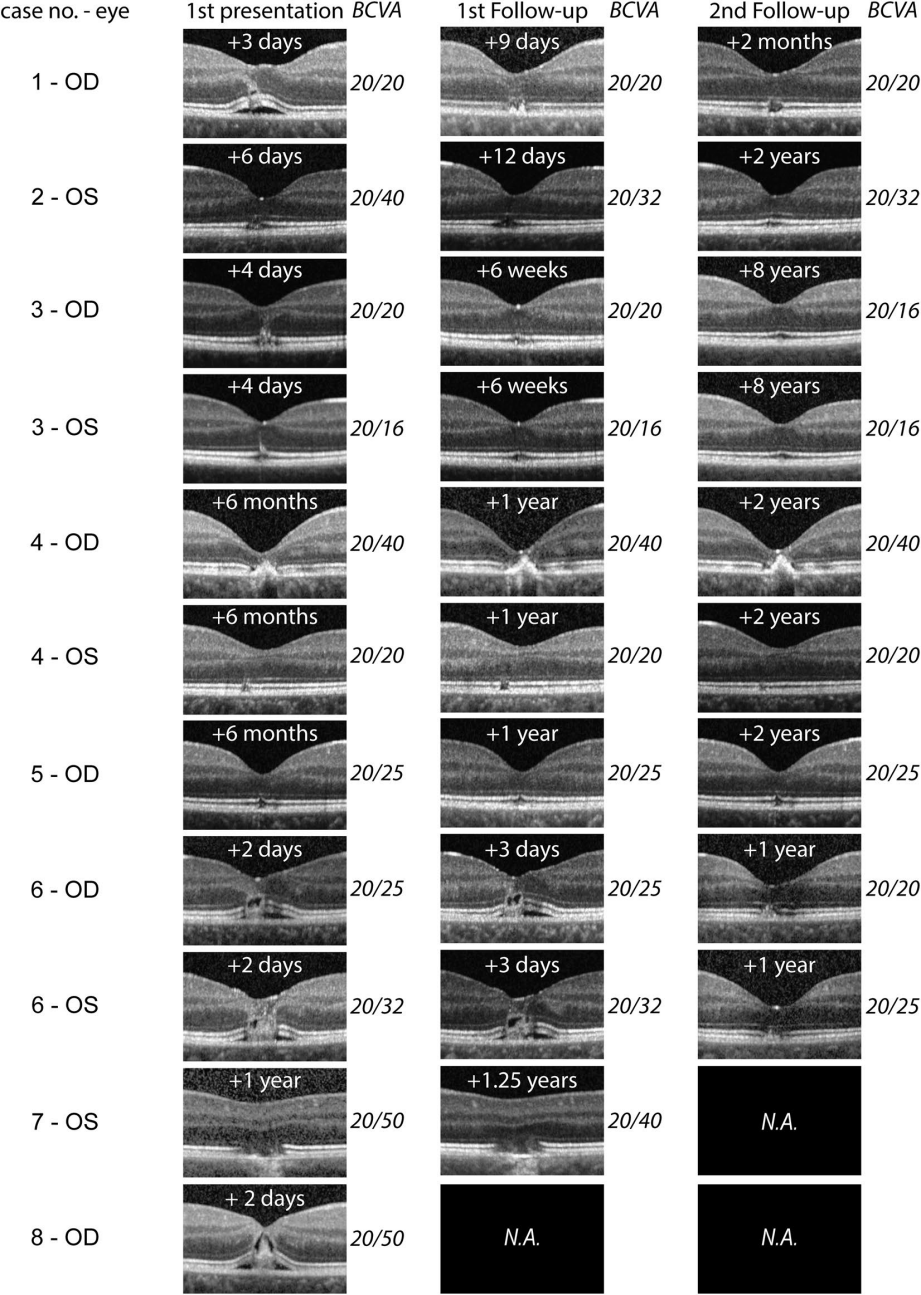
Time course of LPM lesions in macular OCT. Time after injury is indicated at the top of each image. BCVA at the respective time is given on the right of the image

Figure 3 shows the time course of retinal damage and the development of BCVA. In three of eight children (4 of 11 eyes, cases 4, 5 and 7), the initial presentation in our clinic took place more than 6 months after the causative injury, so that the acute course could not be assessed in these children. In the remaining children, the initial presentation was less than a week after the incident. Patient No. 8 did not show up for follow-up examinations.

In the first days after the causative event, sub- and intraretinal fluid was observed in a few cases with extensive damage (case 1 OD, 6 OD, 6 OS, 8 OD). We also saw irregularities of the outer retina with (case 3 OD) or without (case 2 OS) hyperreflective linear streaks. Sub- and intraretinal fluid resolved within days (case 1 OD) whereas outer retinal irregularities such as pigment epithelium clumping (case 1 OD, 4 OD) and especially ellipsoid zone disruption persisted longer (all injured eyes with follow-up), in some cases even for years (case 2 OS, 4 OD, 4 OS, 5 OD, 6 OD, 6 OS). In fundoscopy, lesions did not show marked changes at follow-up compared with initial presentation (data not shown).

### Comparison of laser pointer maculopathy with solar retinopathy

We also identified seven patients (11 eyes) with confirmed solar retinopathy (SR) in the same timeframe (2007–2020). Five patients were male, and two were female. The median age at the time of injury was 32.5 years (range 18–49 years). Four patients were affected bilaterally and three unilaterally. The symptoms of these patients resembled those of patients with LPM: Four complained of a central scotoma and three of blurry vision in the respective eyes. Fundoscopic lesions were unifocal, sometimes ring-shaped and yellowish (data not shown).

We compared the macular OCT phenotypes of solar retinopathy lesions with lesions found in patients with LPM shown before. Online Resource 2 shows macular OCT images of affected eyes at the first and last presentation with the respective BCVAs at each examination. Similar to cases of LPM, we could identify distinct lesion phenotypes depending on the time that had passed after the acute incident. Macular OCTs that were acquired only few days after the causative incident showed hyperreflective linear streaks in the outer retina (case 1 OD, case 4 OU). As in patients with LPM, these hyperreflective streaks fully resolved but disruptions of the outer retina, especially the ellipsoid zone, could persist (cases 1 and 2).

## Discussion

We demonstrate that the number of cases of laser pointer-induced retinal injury has increased in southwestern Germany from 2007 to 2020. Our data also illustrate that the OCT phenotype of laser pointer-induced retinal lesions varies depending on the time passed since injury. In addition, we show that in macular OCT, these lesions strongly resemble lesions found in patients with solar retinopathy.

Concerning time course of the number of patients with LPM, five of eight children were injured in 2019/2020 compared to only three between 2007 and 2018 suggesting an increase in the recent past. On the other hand, the number of cases of SR cases was stable over the same time period (data not shown). In accordance with our data, increasing incidence of LPM has been reported lately [10]–12. The underlying causes remain elusive, but could be attributable to the nonrestrictive legislation regarding the possession and use of higher-power laser pointers in the EU and the US, as well as the easy availability of such devices via the Internet [2].

Patient history may be difficult to obtain since children often do not directly report laser pointer use. Therefore, it is important to look for early retinal signs, which may indicate the past use of a laser pointer. Regarding laser pointer-induced structural retinal damage, our data illustrate that its temporal course is subject to dynamic changes, especially in the first weeks after retinal injury: Acute signs of laser pointer-induced damage in macular OCT include sub- and intraretinal fluid and hyperreflective linear streaks. While these acute signs resolve within days, irregularities or disruptions of the outer retina can persist for long periods of time. Of note, even years after laser pointer-induced retinal damage, we saw disruptions of the ellipsoid zone.

While the spontaneous course is generally positive if the Bruch membrane stays intact and while secondary choroidal neovascularization occurs only in very rare cases [13, 14], the ongoing complaints of patients and the persisting changes in macular OCT even years after the causative incident suggest that some damage may indeed be irreversible [5, 15, 16].

Therapeutic options are limited. Corticosteroids have been proposed for the treatment of laser-induced retinal damage and are used in clinical practice, although their benefit is debatable. Both preclinical and clinical data are partially contradictory and difficult to interpret due to the positive natural course of disease [1, 17, 18]. Our data do not indicate a beneficial effect of oral steroids on the functional outcome in patients with LPM. However, our sample size is small, and therefore, we cannot make any conclusive statement about the effect of oral steroids on the functional outcome after retinal laser pointer injury. Since most, if not all causally related incidents are avoidable, the focus should therefore be to better prevent such laser pointer-induced retinal injuries.

It is known that fixation of the sun can lead to similar injuries [19, 20]. Pathophysiologically, higher power lasers induce photothermal retinal damage within microseconds to seconds, while longer retinal exposure to sunlight leads to photochemical damage [4]. We hence asked whether these pathophysiological differences translate into morphological differences in macular OCT. Patients with LPM and SR shared phenotypic features both in the acute phase and in follow-up examinations. De Silva and colleagues demonstrated that near-infrared reflectance autofluorescence imaging may facilitate discriminating between these disease entities [19]. Other discriminators between these disease entities have been proposed: On the one hand, the age of patients at initial presentation and on the other hand the (multi-)focality of lesions [19, 20]. This is in accordance with our data. While LPM mainly affected children (range in our study: 6–16 years), SR affected patients of all ages (range in our study: 18–49 years). In patients with SR, lesions were unifocal while laser pointers induced multifocal lesions.

Our study has a number of limitations. Of note, the sample size was low due to the monocentric design of the study. Multicentric approaches and meta-analyses/reviews are needed to confirm the notion of increasing incidence of LPM. It has recently been shown that LPM leads to changes in OCT angiography [21] and near-infrared reflectance autofluorescence [19]. Small scotomata often being reported by patients with LPM can be detected by microperimetry. However, due to retrospective nature of the study, these methods were not used in our study. In addition, fundus and macular OCT images were not recorded in a standardized manner. Regarding the temporal course of structural damage in macular OCT, three of eight children did not present to the clinic until 6 months after laser pointer injury. Therefore, the acute course could not be assessed in these children. Also, one child did not appear for follow-up examinations.

In conclusion, our study indicates an increase in cases of LPM in children in recent years. Since LPM is characterized by retinal changes and corresponding symptoms that can persist for years, prevention—especially in vulnerable populations such as children—is of great importance. We therefore recommend that access to laser pointers for children needs to be strictly controlled.

## Supplementary Information

Below is the link to the electronic supplementary material.

Supplementary file1 (TIF 27766 KB)

Supplementary file2 (TIF 90729 KB)

## References

[1] Neffendorf JE, Hildebrand GD, Downes SM (2019). Handheld laser devices and laser-induced retinopathy (LIR) in children: an overview of the literature. Eye.

[2] Hadler J, Tobares E, Dowell M (2013). Random testing reveals excessive power in commercial laser pointers. J Laser Appl.

[3] Spelsberg H, Hering P, Reinhard T, Sundmacher R (2000). Bilateral scleral thermal injury: complication after skin laser resurfacing. Arch Ophthalmol.

[4] Barkana Y, Belkin M (2000). Laser eye injuries. Surv Ophthalmol.

[5] Birtel J, Hildebrand GD, Issa PC (2020). Laser pointer: a possible risk for the retina. Klin Monbl Augenheilkd.

[6] American National Standards Institute (2014) ANSI Z136.1 (2014)–Safe Use of Lasers. USA

[7] Official Journal of the European Union (2014) Commission decision of 5 February 2014 on the safety requirements to be met by European standards for consumer laser products pursuant to directive 2001/95/EC of the European Parliament and of the Council on general product sa. European Union. pp. 20–21

[8] International Electrotechnical Commission (2014) IEC60825–1 2014. In: Safety of laser products: part 1—equipment classification and requirements. Geneva

[9] Bundesanstalt für Arbeitsschutz und Arbeitsmedizin (2013) Technische spezifikation zu lasern als bzw. in verbraucherprodukte(n). Germany

[10] Torp-Pedersen T (2018). Laser pointer maculopathy—on the rise?. Acta Ophthalmol.

[11] Bhavsar KV, Michel Z, Greenwald M, Cunningham ET, Freund KB (2021). Retinal injury from handheld lasers: a review. Surv Ophthalmol.

[12] Marshall J, O’Hagan JB, Tyrer JR (2016). Eye hazards of laser ‘pointers’ in perspective. Br J Ophthalmol.

[13] Chen X, Dajani OAW, Alibhai AY, Duker JS, Baumal CR (2021). Long-Term Visual Recovery in Bilateral Handheld Laser Pointer-Induced Maculopathy. Retin Cases Brief Rep.

[14] Tran K, Wang D, Scharf J, Sadda S, Sarraf D (2020). Inner choroidal ischaemia and CNV due to handheld laser-induced maculopathy: a case report and review. Eye (Lond).

[15] Lee GD, Baumal CR, Lally D, Pitcher JD, Vander J, Duker JS (2014). Retinal injury after inadvertent handheld laser exposure. Retina.

[16] Hossein M, Bonyadi J, Soheilian R, Soheilian M, Peyman GA (2011). SD-OCT features of laser pointer maculopathy before and after systemic corticosteroid therapy. Ophthal Surg Lasers Imaging Retin.

[17] Brown J, Hacker H, Schuschereba ST, Zwick H, Lund DJ, Stuck BE (2007). Steroidal and nonsteroidal antiinflammatory medications can improve photoreceptor survival after laser retinal photocoagulation. Ophthalmology.

[18] Schuschereba ST (1999). High-dose methylprednisolone treatment of laser-induced retinal injury exacerbates acute inflammation and long-term scarring. Ophthal Technol.

[19] De Silva SR (2019). Improved diagnosis of retinal laser injuries using near-infrared autofluorescence. Am J Ophthalmol.

[20] Ortiz Salvador M, Montero Hernández J, Castro Navarro V (2020). Multimodal imaging in laser pointer maculopathy. Arch Soc Esp Oftalmol.

[21] Tomasso L (2017). Optical coherence tomography angiography findings in laser maculopathy. Eur J Ophthalmol.

