# Supplementary figures and images for: Incidence and long-term outcome of laser pointer maculopathy in children
Source: Int Ophthalmol. 2023 Jan 20;43(7):2397–405. doi: 10.1007/s10792-023-02638-w (PMC10313552; doi:10.1007/s10792-023-02638-w)

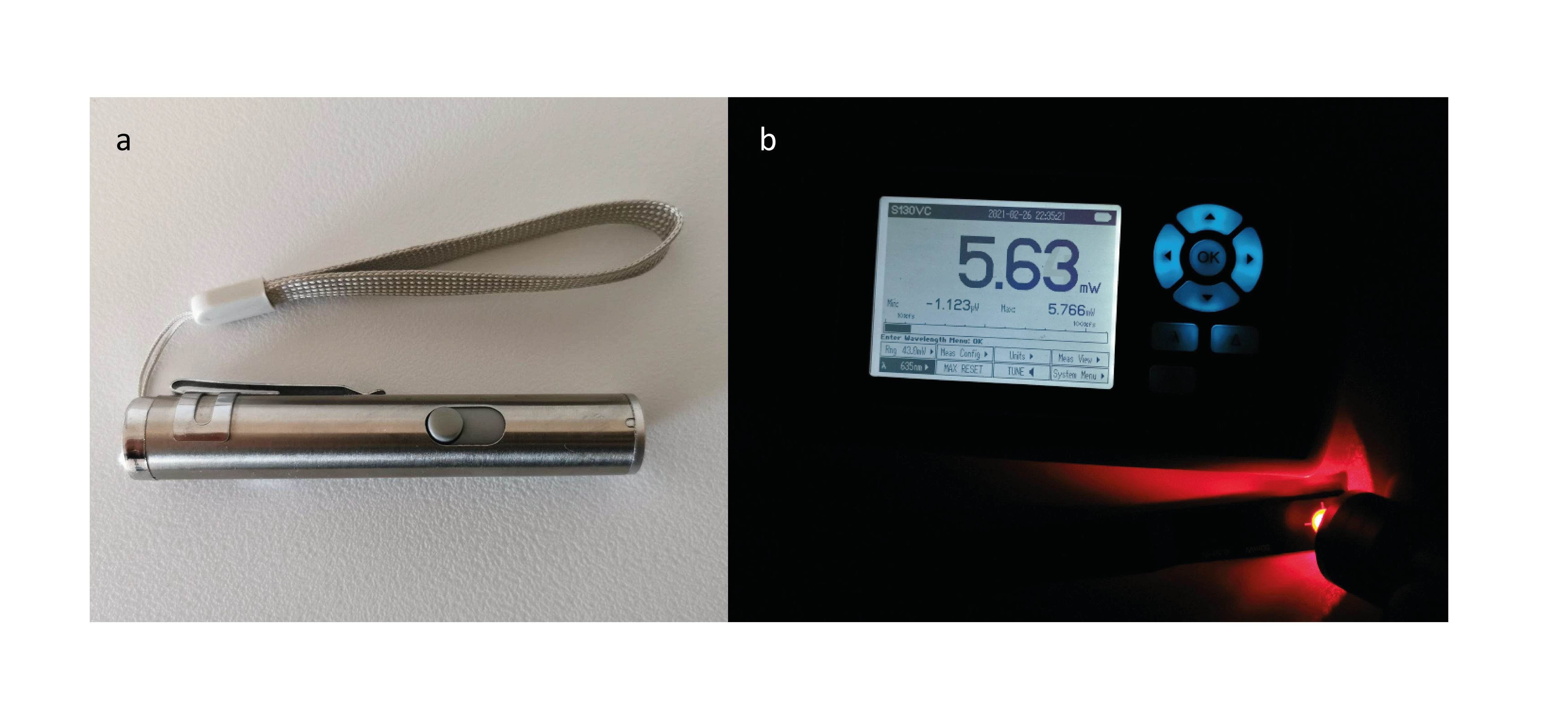

Supplement: Supplementary file 1 — Supplementary file1 (TIF 27766 KB) [file 10792_2023_2638_MOESM1_ESM.tif]
